# Enhancing the Activity of Carboxymethyl Cellulase Enzyme Using Highly Stable Selenium Nanoparticles Biosynthesized by *Bacillus paralicheniformis* Y4

**DOI:** 10.3390/molecules27144585

**Published:** 2022-07-18

**Authors:** Yidan Wang, Yonghe Yu, Yuhua Duan, Qin Wang, Xin Cong, Yi He, Chao Gao, Muhammad Hafeez, Saad Jan, Syed Majid Rasheed, Shuiyuan Cheng, Zhangqian Wang

**Affiliations:** 1 National R&D Center for Se-Rich Agricultural Products Processing, School of Modern Industry for Selenium Science and Engineering, Wuhan Polytechnic University, Wuhan 430023, China; wangyd199907@163.com (Y.W.); yyh13169330283@gmail.com (Y.Y.); dyhrogen@163.com (Y.D.); yi.he@whpu.edu.cn (Y.H.); gaochao@whpu.edu.cn (C.G.); 2 State Key Laboratory of Biocatalysis and Enzyme Engineering, School of Life Sciences, Hubei University, Wuhan 430062, China; qin.wang@hubu.edu.cn; 3 Enshi Se-Run Health Tech Development Co., Ltd., Enshi 445000, China; 13905189777@163.com; 4 State Key Laboratory of Rice Biology, Ministry of Agriculture Key Lab of Molecular Biology of Crop Pathogens and Insects, Institute of Insect Sciences, Zhejiang University, Hangzhou 310058, China; hafeez_203@yahoo.com; 5 Department of Agriculture Entomology Section, Bacha Khan University, Charsadda KP 39250, Pakistan; drsaadjan@bkuc.edu.pk (S.J.); smrasheed@bkuc.edu.pk (S.M.R.)

**Keywords:** selenium enrichment, *Bacillus paralicheniformis*, selenium nanoparticles (SeNPs), carboxymethyl cellulase (CMCase)

## Abstract

The inorganic selenium is absorbed and utilized inefficiently, and the range between toxicity and demand is narrow, so the application is strictly limited. Selenium nanoparticles have higher bioactivity and biosafety properties, including increased antioxidant and anticancer properties. Thus, producing and applying eco-friendly, non-toxic selenium nanoparticles in feed additives is crucial. *Bacillus paralicheniformis* Y4 was investigated for its potential ability to produce selenium nanoparticles and the activity of carboxymethyl cellulases. The selenium nanoparticles were characterized using zeta potential analyses, Fourier transform infrared (FTIR) spectroscopy, and scanning electron microscopy (SEM). Additionally, evaluations of the anti-α-glucosidase activity and the antioxidant activity of the selenium nanoparticles and the ethyl acetate extracts of Y4 were conducted. *B. paralicheniformis* Y4 exhibited high selenite tolerance of 400 mM and the selenium nanoparticles had an average particle size of 80 nm with a zeta potential value of −35.8 mV at a pH of 7.0, suggesting that the particles are relatively stable against aggregation. After 72 h of incubation with 5 mM selenite, *B. paralicheniformis* Y4 was able to reduce it by 76.4%, yielding red spherical bio-derived selenium nanoparticles and increasing the carboxymethyl cellulase activity by 1.49 times to 8.96 U/mL. For the first time, this study reports that the carboxymethyl cellulase activity of *Bacillus paralicheniforis* was greatly enhanced by selenite. The results also indicated that *B. paralicheniformis* Y4 could be capable of ecologically removing selenite from contaminated sites and has great potential for producing selenium nanoparticles as feed additives to enhance the added value of agricultural products.

## 1. Introduction

Feed is the material basis of modern agriculture and the planting industry. Feed additives can reduce feed costs and improve livestock product quality. However, few kinds of additives can effectively strengthen the nutritional value of feed and increase its utilization rate. The conversion of straw cellulose into feed for livestock and poultry has become a research hotspot. *Bacillus* sp. has been reported to be effective at degrading cellulose [1,2,3]. However, although selenium can significantly improve the nutritional quality of feed, there are few reports on its decomposition of carboxymethyl cellulose (CMC) [4]. Selenium (Se) is one of the essential trace elements for animals, plants, and humans [5,6,7,8]. Selenium occurs mostly in inorganic forms represented by selenate, selenite, and selenide; the first two soluble forms have high bioaccumulation rates and narrow thresholds between the appropriate and toxic concentrations. Most of the organic selenium is found in soil and plants as methyl selenides or selenium amino acids. A chemical or microbial transformation converts inorganic selenium into zero-valent selenium, which is less toxic and insoluble in water [9,10,11]. Red selenium nanoparticles (SeNPs) with a particle size of 50-500 nm exhibit higher activity, improved biocompatibility, and lower cytotoxicity [12,13].

The properties of SeNPs are usually determined by their size, shape, composition, and structure. SeNPs with controllable physical and chemical properties are highly practical. SeNPs are usually synthesized through physical, chemical, and biological methods [9,10,11,14,15]. Furthermore, Selenium nanoparticles have attracted substantial attention recently owing to their great performance, such as their excellent bioavailability and low toxicity. Additionally, while SeNPs have received increasing attention, their application is not optimistic due to their poor stability. To provide feed additives with high absorption efficiency and high utilization rates, it is crucial to produce SeNPs with small particle sizes and high stability.

SeNPs have been reported to be biosynthesized by plants and some microorganisms, making them greener, more economical, and more environmentally friendly [16]. SeNPs synthesized through bacteria, yeasts, and fungi can be the catalyst bed for processing. Microorganisms comprise the predominant strain for producing SeNPs due to their large distribution, ability to withstand extreme environments, rapid growth, and simple structure [17,18,19]. Many microbial groups have been proven to have the ability to synthesize SeNPs, such as *Bacillus* sp. [20], *Lactobacillus* sp. [21], *Pseudomonas aerugosa* [22], *Streptococcus thermophilus* [23], *Burkholderia* sp. [24], etc. Microorganisms, especially *Bacillus* sp., are good chassis cells that secret proteins into their growth medium, which are usually employed for industrial enzyme production [25]. The proteins such as FliC and OmpF that are produced in the bacteria could control the assembly and stability of BioSeNPs [26]. For that reason, we were curious to explore whether the protein-coated SeNPs produced by *B. paralicheniformis* Y4 can promote its stability. Microbial biosynthesis was used to produce SeNPs to combine the advantages of the strain and the SeNPs to achieve increased effects and better applications in feed additives. However, different genera of bacteria have various abilities to synthesize SeNPs on SeO_4_^2−^ or SeO_3_^2−^, which will affect the growth and metabolism of the bacteria to a certain extent. Prior to synthesizing SeNPs, bacteria need to tolerate a certain amount of SeO_4_^2−^ or SeO_3_^2−^. Recent studies have confirmed that *Bacillus* species are widely recognized as useful industrial platform strains, capable of generating a wide range of valuable products, including enzymes and functional proteins [27]. Therefore, *Bacillus* sp. is considered an excellent selenium-resistant bacterium and a capable bacterial resource for the biosynthesis of SeNPs.

Cellulose is extremely difficult to naturally degrade. However, converting straw cellulose into feed for livestock and poultry is crucial [28]. Bacteria are able to degrade cellulose due to their characteristics of producing Carboxymethyl cellulase (CMCase) and rapid growth. The biodegradation of feed with cellulosic decomposing bacteria is not only effective but eco-friendly. The addition of selenium as a trace element to feed can also enhance the nutritional value of the feed, so the enzyme production ability of the strain and the nutritional value of SeNPs can work in conjunction to realize industrialization. More complex glycoside hydrolases provide synergy with higher potency because of the organismal diversity of extreme niches. Most microorganisms have low CMCase activity and poor heat resistance; consequently, cellulose cannot be widely used in the feed and animal husbandry [29]. Hence, environmental protection science and feed enzyme production are increasingly focused on screening bacterial strains with high cellulase activities and high SeNPs conversion efficiency [30].

Therefore, the objective of this study was to investigate the potential of *B. paralicheniformis* Y4, isolated from the soil samples of *Cardamine violifolia*, to reduce selenite into elemental selenium and produce SeNPs as well as observe whether the CMCase activity was increased. The time-course of selenite removal at different concentrations by the isolated strain was monitored. The SeNPs produced during the bacterial reduction of selenite were characterized using zeta potential analyses, Fourier transform infrared (FTIR) spectroscopy, and scanning electron microscopy (SEM). 

## 2. Results

### 2.1. Isolation and Identification of Strains

#### 2.1.1. Determination of Selenium Tolerance of Strains

**Figure 1:**
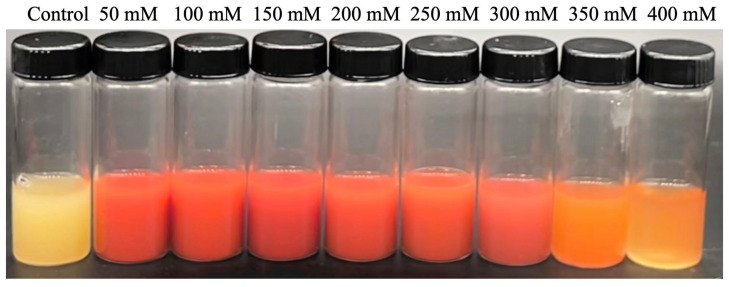
Tolerance of strain Y4 to different concentrations of sodium selenite.

**Figure 2:**
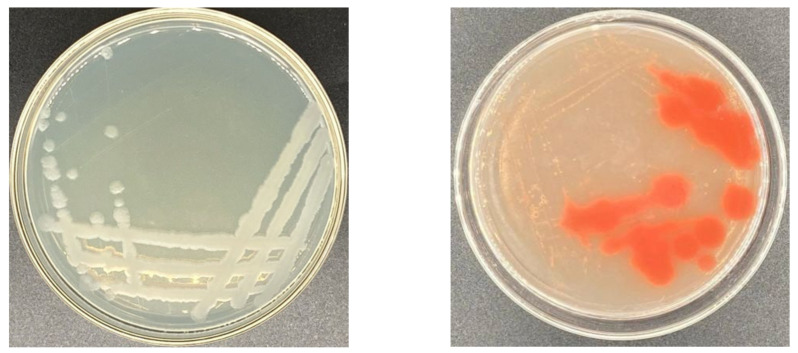
Growth of strain Y4 on Luria–Bertani (LB) agar plates and selenite containing plates. Images of cultures of strain Y4 grown in the absence (**left**) and presence (**right**) of 400 mM selenite. The red colony color indicates selenite reduction and the formation of elemental selenium (Se^0^).

Out of the 58 screened strains, 16 of them were selected for CMCase activity as they were resistant to selenite. Appropriate dilutions of each sample were placed on CMC agar plates. Among the isolated selenite-reducing bacteria, strain Y4 could tolerate 400 mM of selenite and reduce selenite to elemental SeNPs (Figure 1). Additionally, the ratio of primary screening plate hydrolysis circle diameter to pure culture colony diameter (d/D) was 3.23 (Table 1). Previous studies have demonstrated that the color change of the reaction solution from colorless to red indicates the synthesis of SeNPs via selenite oxyanion. The colony was opaque, and the edge of strain Y4 was smooth (Figure 2).

#### 2.1.2. Identification of Strain Y4

The identification results of the physiological and biochemical tests are shown in Table 2. Strain Y4 can utilize mannose and lactose, but not arabinose and sucrose. In the negative indole test and Voges-Proskauer (VP) test, strain Y4 showed the typical characteristics of *Bacillus licheniformis*, which was consistent with the comparison results of *B. licheniformis* in Berger’s manual. 

**Figure 3:**
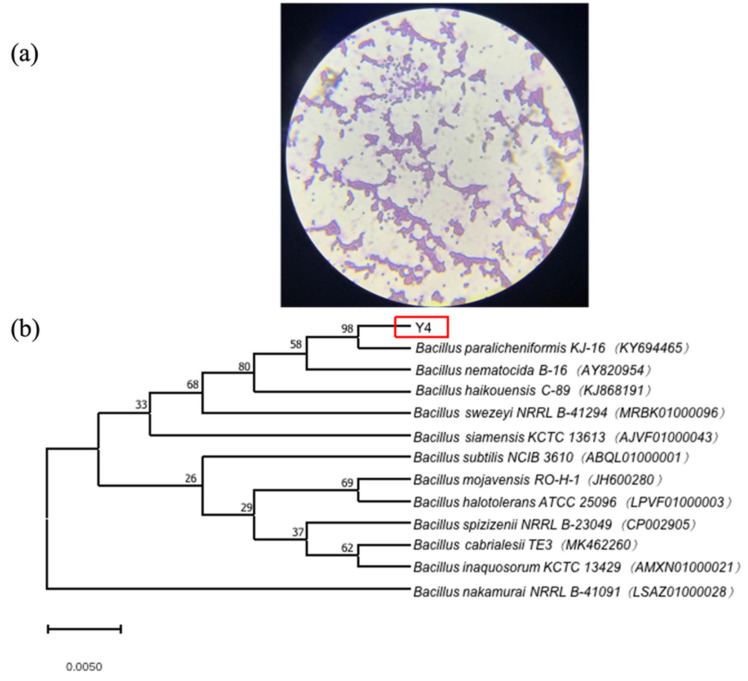
Characterization of strain Y4. Gram staining of strain Y4 (**a**) Neighbour-joining tree inferred through MEGA software based on the sequences of 16S rRNA gene, showing the phylogenetic relationship of strain Y4 and related species obtained from the Ezbiocloud website (**b**).

The morphological and cultural studies revealed that strain Y4 was a rod-shaped, Gram-positive bacterium. The microscopic examination result of the Gram staining is shown in Figure 3. The sequencing of the DNA fragments of strain Y4 provided a complete DNA sequence of the determinant, which was 1500 bp in size. The bacterial ribosomal 16S rRNA was amplified to obtain the corresponding PCR product. Based on the results of Ezbiocloud GenBank, strain Y4 revealed 98% identity in its 16S rRNA gene sequence with respect to *Bacillus paralicheniformis* KJ-16 KY694465. The phylogenetic analyses derived from the neighbor-joining method (a bootstrap method with 1000 repeats) showed the position of strain Y4 among the species of the genus *Bacillus*. Based on the available data, it could be concluded that strain Y4 belongs to *Bacillus paralicheniformis*.

### 2.2. Bacterial Growth in the Presence or Absence of Selenite

**Figure 4:**
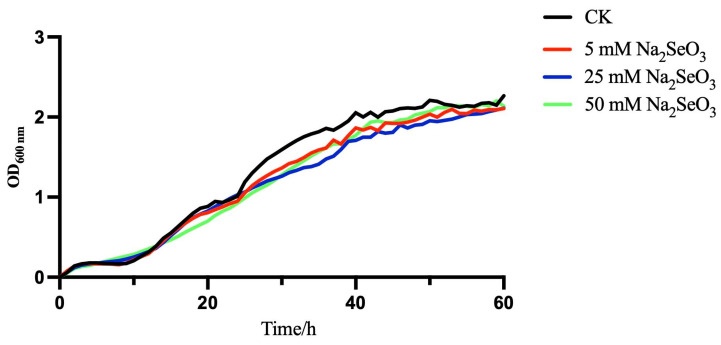
Bacterial growth of strain Y4 supplied with 0, 5, 25, and 50 mM Na_2_SeO_3_.

The growth of *B. paralicheniformis* Y4 in LB was monitored over time in a 96-well plate using initial concentrations of selenite of 0, 5, 25, and 50 mM after 60 h, which were drawn by the automatic growth curve analyzer (Bioscreen C MBR., Turku, Finland) in Figure 4. Strain Y4 was adapting to the new cultural environment (LB containing 0-, 5-, 25-, and 50-mM selenite) and entered the lag phase at 0–10 h; due to the slow bacterial reproduction, the curve was flat and stationary. In the presence or absence of selenite, the bacteria metabolized vigorously and the absorbance value at 600 nm increased significantly, which indicated the logarithmic phase at approximately 10 h. At 50 h, the absorbance value of the bacterial solutions was kept stationary, and *B. paralicheniformis* Y4 entered the stationary phase, which has a similar tendency in the literature [31]. The results showed that *B. paralicheniformis* Y4 could normally grow in those four different environments, even in the initial concentration of selenite at 50 mM, indicating that the strain could be a sort of selenium-tolerant bacteria. Furthermore, the addition of sodium selenite at different concentrations did not significantly affect the lag phase of the strain. 

### 2.3. Selenite Reduction by B. paralicheniformis Y4

Previous research has shown that a low concentration of sodium selenite could promote plant growth while a high concentration of sodium selenite may have toxic effects on plants [32,33,34]. Therefore, selecting an appropriate concentration of sodium selenite is crucial. Many of the researchers from the literature selected the concentration range of 0–10 mM [35,36,37], and our experiments also found that the lower the concentration of sodium selenite, the smaller the impact on the growth of bacteria, and the faster the reduction rate. Therefore, a concentration of 5 mM was selected to explore the selenite reduction rate and the following experiments.

**Figure 5:**
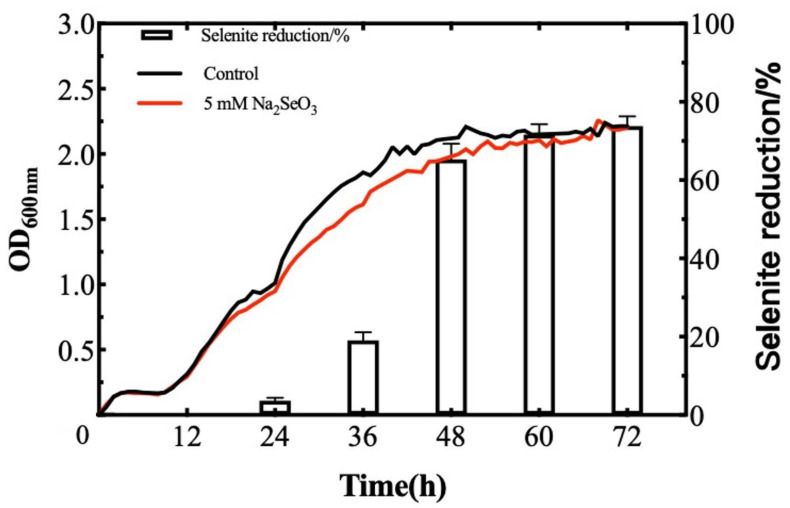
Selenite reduction rate and bacterial growth by strain Y4 at a supplied selenite concentration of 5 mM in LB broth after incubation at 37 °C under the agitation of 180 rpm. Values are mean ± SD of three replicates.

Currently, reports on bacteria with a high tolerance to selenite and a fast reduction rate are scarce, which somewhat limits the utilization of SeNPs in actual production. Therefore, selenite-tolerant strains must be screened as well as strains with a high transformation rate for SeNPs production. The reduction of selenite and the formation of the SeNPs by *B. paralicheniformis* Y4 was a continuous process related to the growth of the strain. When 5 mM SeO_3_^2−^ was supplied in the medium, the reduction process started concomitantly with the onset of the microbial growth. After 6 h, the cell culture began to turn red, indicating the reduction of SeO_3_^2−^ into Se^0^. A total of 76.4% of SeO_3_^2−^ was reduced to Se^0^ when the incubation continued for 72 h (Figure 5). 

### 2.4. Characterization of the SeNPs Produced by B. paralicheniformis Y4

#### 2.4.1. SEM Analysis

An excessive concentration of sodium selenite may destroy the integrity of strain cells [38]. To clearly observe the distribution positions of the bacteria and SeNPs under a high concentration of sodium selenite, 50 mM of selenite (10 times the selenite reduction testing concentration) was selected to determine whether this high concentration would affect the integrity of strain Y4 cells. SeNPs in the presence of selenite at 50 mM inoculated with strain Y4 were collected which revealed spherical structures associated with the cellular biomass analyzed by scanning electron microscopy (SEM). 

**Figure 6:**
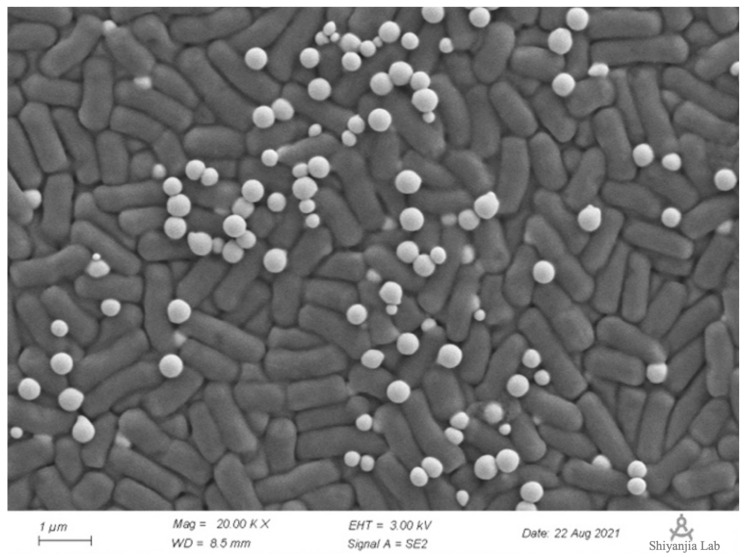
Scanning electron microscopy (SEM): strain Y4 with 50 mM selenite after 24 h of incubation.

As Figure 6 shows, SeNPs produced by *B. paralicheniformis* Y4 could be found individually attached to the outer cell membrane or as agglomerates around bacterial cells in the presence of selenite. Despite the extensive accumulation of the SeNPs with a tendency to form aggregates of individual particles, no evidence of cell lysis or the distortion of the outer membrane was observed [39,40]. The particles appeared to have a spherical shape and a diameter of approximately 70–120 nm with an average of 80 nm, which has a uniform shape and good dispersion [15]. Furthermore, strain Y4 was able to reduce the toxic selenite aerobically with the concomitant generation of the SeNPs in LB at a logarithmic phase, as Figure 6 showed. 

#### 2.4.2. Fourier Transform Infrared (FTIR) Spectroscopy Analysis

**Figure 7:**
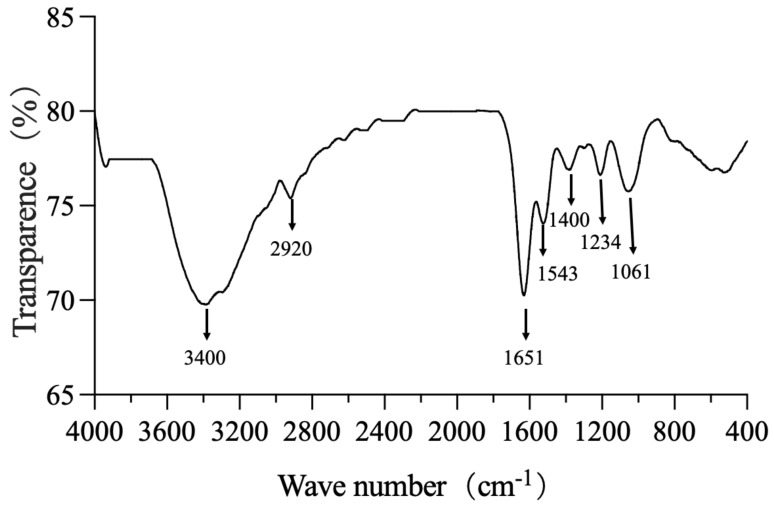
The 4000–400 cm^−1^ FTIR spectrum of SeNPs synthesized by strain Y4.

By measuring the vibrational frequencies in the chemical functional groups, the FTIR spectroscopic technique revealed the functional groups on the surface of the SeNPs. The groups such as O-H, CH2, CH3, C=O, and C-O-C existing on the surface of the biogenic SeNPs produced by *B. paralicheniformis* Y4 may be coated by proteins and polysaccharides that were involved in the formation and stabilization of the SeNPs (Figure 7). The various absorption bands and corresponding distributions in the FTIR spectrum were as follows: the band at 3400 cm^−1^ was the stretching vibration of the O-H group. The peak centered at 2920 cm^−1^ (broad and asymmetric) was a sum of the various stretching vibrations of the C-H moieties in the methyl and methylene groups, largely in protein side chains, while the peak at 1651 cm^−1^ indicated the presence of C=O (amide I band) in the amide group [41]. The peak at 1543 cm^−1^ corresponded to the N-H plane bending of protein amide II. The weak and broad peak centered at 1400 cm^−1^ evidently contains contributions from various vibrations such as the stretching of C-N, the bending of C-H, and the symmetric stretching modes of ionized carboxylic groups. The peak at 1234 cm^−1^ corresponded to amide III and to asymmetric stretching vibrations of the O–P=O moieties that are typical for proteins. The broad peak at 1061 cm^−1^ is within the typical region of C-O/C-C/C-N vibration modes characteristic of polysaccharides and/or proteins. Hence, FTIR spectroscopy (particularly the typical amide I and II bands) clearly showed the presence of proteins and probable polysaccharides on the surface of the obtained biogenic SeNPs [41,42]. The FTIR spectra clearly showed organic residues such as carbohydrates and proteins on the surface and probable polysaccharides of the SeNPs produced by *B. paralicheniformis* Y4. 

### 2.5. The Effect of pH on the Stability of SeNPs

**Figure 8:**
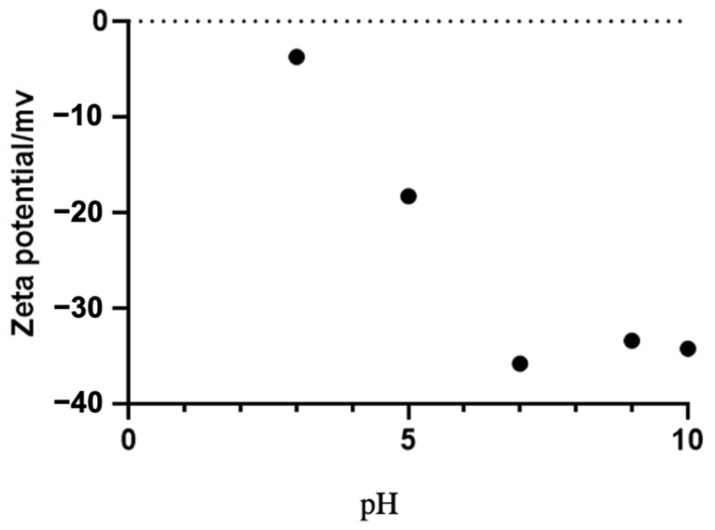
Effect of different pH on zeta potential of SeNPs.

The zeta potential measures a reasonable electric charge on the surfaces of nanoparticles. The nanoparticles with a higher magnitude of zeta potential exhibit an increased stability due to the greater electrostatic repulsion between the nanoparticles. The stability of the SeNPs was confirmed by the measurement of the zeta potential in a wide pH range. As shown in Figure 8, when the pH was 7–9, the zeta potential value of the SeNPs was below −30 mV.

**Figure 9:**
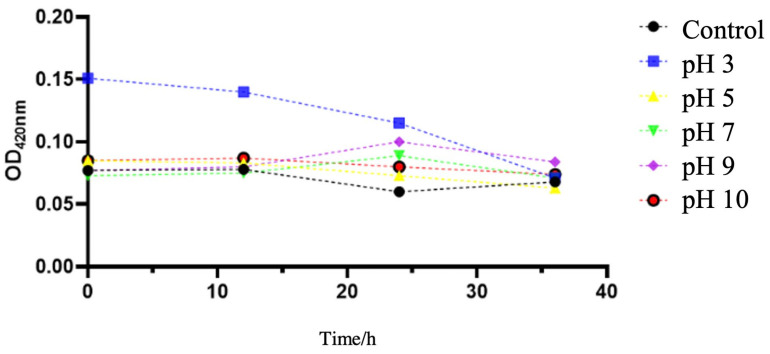
Effect of different pH on sedimentation of SeNPs.

The results of the measurement of the sedimentation rate of the SeNPs were observed under dynamic conditions in a wide pH range (Figure 9). As the reaction time progressed, the overall absorbance of the solution decreased. At a pH of 7, the absorbance of the SeNPs solutions was stable, suggesting that the SeNPs were uniformly shaped and stable at this pH level. The stability of the SeNPs at pH 7-9 was high, which was consistent with the trend of the potential measurement results.

### 2.6. Evaluation of Probiotic Properties of B. paralicheniformis Y4

#### 2.6.1. α-Glucosidase Inhibitory Assay

**Figure 10:**
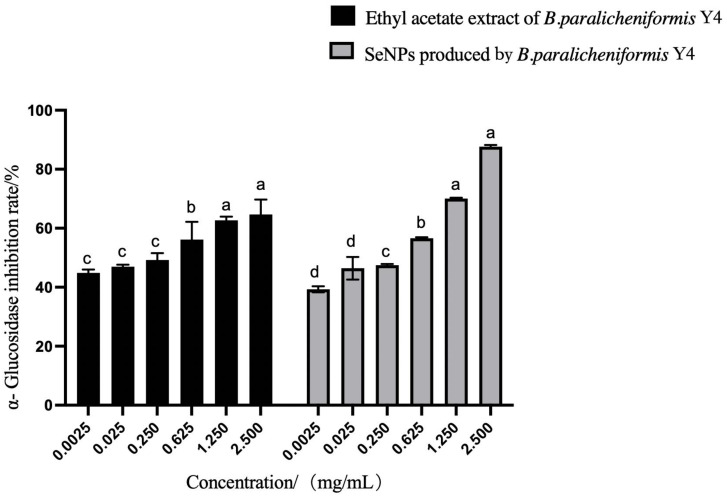
The inhibition rate for a-glucosidase of SeNPs and ethyl acetate extract of strain Y4. The different alphabetic superscripts in the same column are significantly different (*p* < 0.05) based on Tukey’s multiple comparison test.

The α-glucosidase inhibitory activity of the SeNPs and the ethyl acetate extract of *B. paralicheniformis* Y4 showed a certain inhibitory activity, as shown in Figure 10. The inhibition rate of α-glucosidase was directly proportional to the concentration of the ethyl acetate extract of *B. paralicheniformis* Y4, and the maximum inhibition rate was 78.5% at 2.50 mg/mL.

#### 2.6.2. Antioxidant Evaluation of Ethyl Acetate Extract

The antioxidant molecules quench 2,2-Diphenyl-1-picrylhydrazyl (DPPH) radicals (by providing hydrogen atoms or electron donations) and convert them into colorless products. The 2,2’-Azino-bis-3-ethylbenzthiazoline-6-sulfonate (ABTS) decolorization assay is a rapid and reliable method that is widely used in the total radical scavenging measurement of the strain extracts. The evaluation of the free radical scavenging ability of *B. paralicheniformis* Y4 is shown in Table 3. 

The free radical scavenging ability increased with the concentration of the ethyl acetate extract of *B. paralicheniformis* Y4. The ethyl acetate extract of strain Y4 had a remarkable free radical scavenging rate of 98.8% at 16 μg/mL; at 480 μg/mL, the DPPH free radical scavenging rate reached 33.1%, and the hydroxyl free radical scavenging rate at 320 μg/mL reached 80.7%.

### 2.7. Environmental Adaptability of Strain Y4

#### 2.7.1. Effects of Different Temperatures on Strain Growth

**Figure 11:**
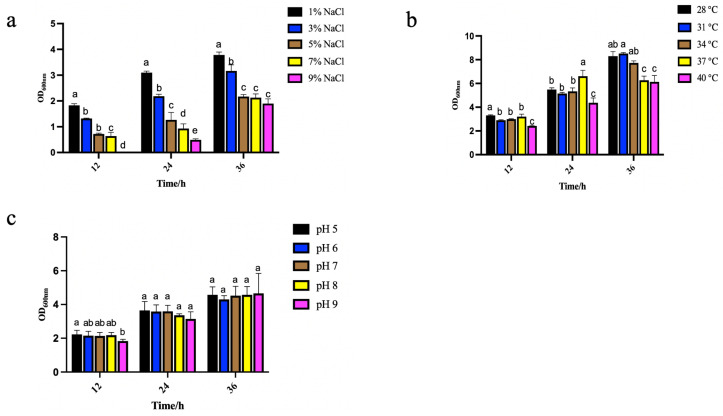
Effects of different temperatures (**a**), NaCl concentrations (**b**), and pH (**c**) on the growth of strain Y4. The different alphabetic superscripts in the same column are significantly different (*p* < 0.05) based on Tukey’s multiple comparison test.

The effect of different culture temperatures on the growth of *B. paralicheniformis* Y4 is shown in Figure 11a. The culture temperature remarkably affected the microorganisms’ growth, reproduction, and physiological metabolism. In the fermentation process, the influence of the temperature was mainly reflected in microbial growth and reproduction, metabolic synthesis, the physical and chemical properties of the fermentation broth, etc. [43]. As it is well known, temperature control and regulation have a great impact on industrial production. So, while comprehensively considering the cost and efficiency, we demonstrated that *B. paralicheniformis* Y4 reached the best growth state at 37 °C for 24 h.

#### 2.7.2. Effects of Different NaCl Concentrations on Strain Growth

Figure 11b shows the effect of different NaCl concentrations on the growth of *B. paralicheniformis* Y4 when the temperature was set to 37 °C. NaCl in high concentrations within the culture medium will increase the osmotic pressure of the solution, the imbalance of osmotic pressure in microbial cells, and cause cell death due to the water shortage. *B. paralicheniformis* Y4 was in a great growth state when the culture temperature was 37 °C and the NaCl concentration was 1%, which corresponds to a similar trend in the literature [44,45]. Therefore, a NaCl concentration at 1% was selected for the following experiment.

#### 2.7.3. Effects of Different pH on Strain Growth

Figure 11c shows the effect of a different pH on the growth of *B. paralicheniformis* Y4 when the temperature and the NaCl concentration were set at 37 °C and 1%. The pH influenced the development of microorganisms. Particularly, the change of the cell membrane’s surface charge may affect the absorption of nutrients by microorganisms [46]. By observing the growth performance, it was demonstrated that the cell culturing at a pH of 5.0 to pH 9.0 had little effect on the growth of bacteria at different periods, as shown by measuring the OD_600nm_. 

### 2.8. CMCase Activity Assay Containing Selenite

**Figure 12:**
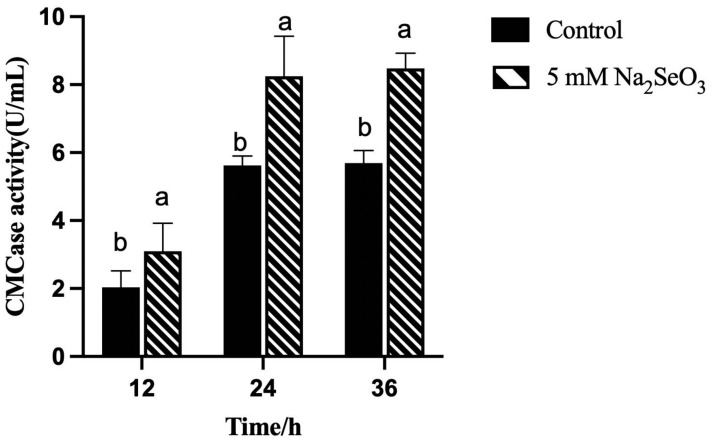
CMCase activity was produced by culture medium containing different concentrations of selenite at 37 °C after 24, 48, and 72 h by *B. paralicheniformis* Y4. The different alphabetic superscripts in the same column are significantly different (*p* < 0.05) based on Tukey’s multiple comparison test.

The results of the CMCase activity produced by *B. paralicheniformis* Y4 after 24, 48, and 72 h with 5 mM selenite and without adding selenite are shown in Figure 12. The CMCase activity was gradually increased with time in the presence of 5 mM selenite. The enzyme activity increased sharply up to 72 h, and the highest CMCase activity of 8.96 U/mL was 1.49-fold better than that of the control. The increase in the CMCase activity might be due to the reduction of selenite by the *B. paralicheniformis* Y4 to produce SeNPs or some intermediate metabolites, which then stimulated the degradation of CMC.

## 3. Discussion

The results have suggested that the maximum selenium tolerance concentration of strain Y4 can reach 400 mM, enabling further studies on biological SeNPs, while the selenium tolerance of *Bacillus* is generally within a range of 1-250 mM [47,48]. Dhanjal et al. screened a *Bacillus cereus* CM100B strain from coal mine soil, which could tolerate 10 mM selenite [35]. Asad Ullah et al. screened a strain of *Bacillus subtilis* BSN313 from Chinese koji with a maximum tolerance to selenite of 2.5 mM, which is far lower than strain Y4, indicating that *B. paralicheniformis* Y4 could be applied as a selenium-tolerant strain [49].

Then, we evaluated the profiles of the bacterial growth containing selenite and without selenite. As the results have shown, the addition of sodium selenite at different concentrations did not significantly affect the lag phase of the strain, which is beneficial for the growth of strain Y4. However, the phenomenon observed in the studies showed that after a 2 h-inoculation, *Bacillus subtilis* BSN313 entered the logarithmic and stable phases at 16 h. After 22 h, it entered the death stage. As for *Bacillus paramycoides* SP3, the major depletion of SeO_3_^2−^ shifted from the exponential to the stationary phase with an increasing initial concentration [49,50]. The treatment group was supplemented with selenite, whereas the control had no selenite. The minimal effect of the three concentrations of selenite on the growth of *B. paralicheniformis* Y4 revealed the high tolerance of this strain to selenite. After 72 h of incubation with 5 mM selenite, *B. paralicheniformis* Y4 was able to reduce the SeO_3_^2−^ concentration by 76.4%, yielding red spherical bio-derived selenium nanoparticles. Since the reduction process started in conjunction with the onset of the microbial growth, it may be speculated that the reduced tolerance of *B. paralicheniformis* Y4 to selenite is related to the initial addition of selenite or the growth stage of bacteria. Zhao et al. found that when setting the initial concentration of SeO_3_^2−^ at 5 mM, *B. mirabilis* YC801 could only reduce 2.4% of SeO_3_^2−^ within 12 h [51]. A similar phenomenon has also been reported in the literature. When a culture of *Alcaligenes faecalis* Se03 was added with 5 mM SeO_3_^2−^, 73% of the reduction of SeO_3_^2−^ occurred at the end of the plateau stage and the stable stage [40]. The delayed production of elemental selenium was also found in *B. fungorum* DBT1 in NB medium containing 2 mM SeO_3_^2−^. The research indicated that SeO_3_^2−^ may be transformed into the intermediate reduced selenium form of organic selenide, resulting in the delay of the formation of elemental selenium [50]. Thus, the 76.4% reduction of SeO_3_^2−^ by *B. paralicheniformis* Y4 within 72 h may be attributed to inorganic and organic forms of selenium, such as hydrogen selenide (H_2_Se) that exists in gaseous form [52].

The selenium nanoparticles were characterized, and the particle size was approximately 70–120 nm with an average of 80 nm as determined by our analysis, which is consistent with that calculated for biologically generated SeNPs using various bacterial strains. Several *Bacillus* species have been found to produce SeNPs from 80–400 nm, such as *Bacillus subtilis* [53] and *Bacillus* sp. MSh-1 [54]. The average particle size of 80 nm and the good dispersion of the SeNPs produced by *B. paralicheniformis* Y4 made increased their activity and indicated their promising broader applications. FTIR spectroscopy was used to determine the functional groups responsible for the nanoparticle synthesis by *B. paralicheniformis* Y4. The results showed the functional groups on the surface of the biosynthesized SeNPs, which were previously recorded in the literature, such as *Stenotrophomonas maltophilia* [55], *Thauera sellenatis* [56], and *Rahnella aquatilis* HX2 [39]. The above studies indicated that the proteins surrounding SeNPs of *B. paralicheniformis* Y4 take part in reducing selenite, as well as synthesizing and stabilizing SeNPs.

Furthermore, the high negative charge on the surface of the nanoparticles could indicate a high degree of stability for biogenic nanoparticles without any processing or treatment, e.g., adding dispersants such as starch. We have demonstrated that SeNPs are electrokinetically stable in a wide pH range, with the zeta potential reaching −35.8 mV at a pH of 7, which is a range of great stability for application [35,49,50,56]. The determination of the sedimentation rate of the SeNPs is also crucial for determining their stability. Considering the result of the zeta potential and the sedimentation rate, SeNPs produced by *B. paralicheniformis* Y4 are potentially stable and thus well applicable.

Strains with good probiotic properties are crucial as feed additives. The evaluation of the probiotic properties of *B. paralicheniformis* Y4 showed that *B. paralicheniformis* Y4 could not only reduce selenite to SeNPs but also had a certain α-glucosidase inhibitory activity that may be used as a potential auxiliary hypoglycemic strain. The ethyl acetate extract of *B. paralicheniformis* Y4 was also recorded; it had certain DPPH free radical scavenging ability and hydroxyl free radical scavenging ability and showed high free radical scavenging activities in ABTS, indicating a good probiotic activity.

Feed additives need excellent absorption efficiency and high stability, so the ability of strains to adapt skillfully to the environment is essential. When the culture temperature was 28–40 °C, *B. paralicheniformis* Y4 was in good condition, reproduced, and played the role of selenium pollution repair. When the concentration of NaCl was 1–3%, *B. paralicheniformis* Y4 could grow well, which was conducive to the production and application of *B. paralicheniformis* Y4. Furthermore, the cell culturing at pH 5.0 to 9.0 had little effect on the growth of bacteria at different periods. Many selenium-containing wastewaters are oil refining or mineral treatment wastewaters. Their pH value is acidic or slightly acidic, which is considerable for applying *B. paralicheniformis* Y4 to treat polluted selenium environments, especially selenium-containing wastewater. *B. licheniformis* is used commercially for feed applications or plant biological protection, and these applications do not require the attainment of antimicrobial resistance genes that may be transmitted to pathogens. *Bacillus licheniformis* Y4 has potential probiotic properties and can also reduce sodium selenite to produce SeNPs with beneficial advantages, and the combination of the strain and SeNPs can be better applied in feed. [57,58]. The growth-promoting potential of probiotics is realized once they have entered the digestive tracts of animals. *B. paralicheniformis* Y4 was in good condition under a range of temperatures (28–37 °C), NaCl concentrations (1–3%), and pH (5–9), which may be used as feed additives and for the production of Se(0)-containing industrial products.

The carboxymethyl cellulase activity increased by 1.49 times to 8.96 U/mL compared to the control group. Among the metal ions, Na^+^, Ca^2+^, and Fe^2+^ stimulated the CMCase activity while selenium’s effect towards it is rarely reported, which is reflected in our interesting and promising finding [59]. The same phenomenon can also be observed by increasing CMCase activity under the external stimulation of other substances. The CMCase activity was enhanced with an increase in the sulfide concentration. In conclusion, sulfide enhanced the degradation of both amorphous and crystalline substrates. As a result, it may be added to a variety of industrial applications to improve CMC digestion [60]. The enzyme activity was significantly improved after optimizing the culture temperature, carbon, the nitrogen source concentration, pH, and the rotating speed [61]. Sreena CP et al. found that the strain’s cellulase activity was different in different media, so optimizing the enzyme production conditions was the key to improving the cellulose degradation rate [62]. Kato et al. found that the efficiency of the strain was significantly lower than that of mixed strains when cultured alone for lignocellulose degradation [63]. Therefore, we can further optimize culture conditions, co-culture *B. paralicheniformis* Y4 with other strong enzyme production ability strains, to improve the cellulose degradation ability so that *B. paralicheniformis* Y4 can be used as a new SeNPs feed additive and realize its comprehensive application. 

## 4. Materials and Methods

### 4.1. Supplies and Chemicals

Yeast powder, Selenite, Glucose, Sodium chloride, Sodium hydroxide, Potassium dihydrogen phosphate, Potassium dihydrogen phosphate, Potassium dihydrogen phosphate, Potassium dihydrogen phosphate, Nitric acid, α-D-glucoside, Peptone, and α-glucosidase were purchased from Oxoid in France. Biological grade agar and carboxymethyl cellulose (CMC-Na) were purchased from Sigma-Aldrich. 2,2-Diphenyl-1-picrylhydrazyl (DPPH, ≥95.0%), 2,2’-Azino-bis-3-ethylbenzthiazoline-6-sulfonate (ABTS, ≥95.0%),Salicylic acid, and FeSO_4_ were purchased from Sinopharm Chemical Reagent Co., Ltd., Shanghai, China.

### 4.2. Isolation of the Strain with High Reduction of Na_2_SeO_3_ and CMCase Activity

The soil samples were collected from a *Cardamine violifolia* (the only selenium hyperaccumulation plant found in China) field at a depth of 10 cm in Enshi (latitude 30°39′ N, longitude 109°48′ E), Hubei Province [64,65,66]. Strains were isolated using a repeated enrichment technique at 37 °C for 24 h in 200 mM selenite (Na_2_SeO_3_) supplemented with LB culture. Red coloration indicates the reduction of selenite to elemental selenium. The capability of the screened strains to transform selenite to elemental selenium was tested in LB liquid medium at 50-, 100-, 150-, 200-, 250-, 300-, 350-, and 400-mM concentrations of Na_2_SeO_3_ incubated at 37 °C under the agitation of 180 rpm for 48 h. The precipitation amount and color change of the bacteria were observed. The 16 strains, observed to be tolerant of up to 200 mM selenite, were selected to determine carboxymethyl cellulase activity (CMCase).

A total of 16 strains were spread onto carboxymethyl cellulose (CMC) agar plates, and then incubated at 37 °C for 16 h. The plates were prepared separately for staining and flooded with Congo red for 20 min, and then washed with 1 M NaCl. Strain Y4 was selected for the highest CMCase activity combined with selenium tolerance.

### 4.3. Identification of the Strain

#### 4.3.1. Morphological Identification of Strain Y4 Containing Selenite

To evaluate the selenite tolerance, strain Y4 was grown in LB containing 400 mM of selenite, and crystal violet staining was optimized at × 100 for microscopic observation of cell morphology. 

#### 4.3.2. Taxonomical Analyses of Strain Y4

The phenotypic and molecular characterization of strain Y4 was performed based on the methods described in Berger’s Manual of Determinative Bacteriology and 16S rRNA gene sequence analysis [67,68]. The 16S rDNA gene was amplified through PCR using 27F/1492R primer sets. Conditions for 16S rDNA gene amplification were as follows: 95 °C for 5 min, 30 cycles of denaturation at 94 °C for 1 min, annealing at 55 °C for 1 min, extension at 72 °C for 90 s, followed by a final extension at 72 °C for 10 min. 

The 16S rDNA sequence of strain Y4 was compared with other sequences in the Ezbiocloud database [69]. A phylogenetic tree was constructed using MEGA version 10.0 software based on the neighbor-joining method with a bootstrap of 1000 replications. 

#### 4.3.3. Physiological and Biochemical Tests of Strain Y4

As per the standard bacterial system identification manual, strain Y4 was identified by biochemical, physiological, and molecular analyses. Physiological and biochemical experiments included VP experiment, indole experiment, gelatin liquefaction experiment, and carbon source utilization.

### 4.4. Growth of Strain Y4 in the Presence or Absence of Selenite 

To determine the toxicity of selenite towards strain Y4, the bacterial growth was determined in the presence of 0, 5, 25, and 50 mM of selenite. Growth curve data were generated by culturing in an automated 96-well micro-plate reader at 37 °C with different concentrations of selenite, and photometric readings (optical density: OD 600nm) were taken [65,70,71].

### 4.5. Selenite Reduction by Strain Y4

The literature showed that a low concentration of sodium selenite could promote plant growth while a high concentration of sodium selenite may have toxic effects on plants [32,33,34]. Therefore, the selection of an appropriate concentration of sodium selenite is crucial. Many of the sources in the literature selected the concentration range of 0–10 mM [35,36,37], and our experiments also found that the lower the concentration of sodium selenite, the smaller the impact on the strain OD_600nm_, and the faster the reduction rate. Therefore, we explored the selenite reduction. Strain Y4 was exposed to 5 mM selenite according to the method of Larse E.H. with minor modification by ICP-MS [72].

A 2 mL bacterial culture aliquot was collected after 24, 36, 48, 60, and 72 h. After ultrasonic crushing for 10 min, the sample was centrifuged at 12,000 rpm and 4 °C for 20 min. For residual selenite analysis, the supernatant was filtered through Millipore filters (0.22 μm) and then the supernatant was collected. An aliquot of 1 mL supernatant was transferred into the inner tank, 5 mL nitric acid was added, and these conditions were maintained for 1 h. Then, the temperature in the inner tank was maintained at 55 °C for 30 min.

The ratio of the response signal value of the element was measured, the selected internal standard element was taken as the ordinate, and then the standard curve was drawn. The solutions were injected into the inductively coupled plasma mass spectrometer. The signal response values of the elements and the standard internal elements were measured. The selenium content in the sample and the content of the selenium to be measured were calculated as follows: X = (*ρ* − *ρ*_0_) × V × f/m ∗ 1000.

Where X is the content of elements to be measured in the sample, using units of mg/kg or mg/L; *ρ* is the mass concentration of the measured elements in the sample solution, in micrograms per liter (μg/L); *ρ*_0_ is the mass concentration of the element to be measured in the sample blank, in micrograms per liter (μg/L); V is the constant volume of sample digestive solution, in mL; F is the dilution ratio of the sample; M is the transferred volume of the sample, in milliliters (mL); and 1000 is the conversion factor.

### 4.6. Characterization of SeNPs Synthesized by Strain Y4

#### 4.6.1. Separation and Purification of SeNPs

SeNPs were prepared in 100 mL of LB medium with 50 mM selenite. Strain Y4 inoculum was added with 1 mL (1% *v*/*v*) and grown in a shaking incubator at 37 °C and 180 rpm for 48 h. The red bacterial culture was centrifuged at 10,000 rpm for 30 min, and the pellets were washed with H_2_O 2–3 times. To more adequately break cells and obtain purified SeNPs, liquid nitrogen was added to the pellets for grinding. Then, H_2_O was added and ultrasonicated at 300 W for 10 min. The lower layer was collected after adding 1/5 (*v*/*v*) volume of n-hexane—centrifuged at 10,000 rpm for 30 min [39]. Polyvinyl chloride (PVC; 20, 10, 5, 3, 1.2, 0.8, and 0.22 μm pore sizes) filters were exposed to SeNPs. The pellets were washed with H_2_O 2–3 times. For further characterization, the purified SeNPs were freeze-dried at −40 °C for 24 h according to the modified method of Wang Y et al. [51]. 

#### 4.6.2. Characterization and Morphology of SeNPs 

To identify the major structural groups surrounded by the purified SeNPs, FTIR analysis was performed. Firstly, the freeze-dried KBr was ground into powder and then placed into an infrared dryer for drying. Then, 1 mg SeNPs was mixed evenly with KBr (100 mg; 200 mesh) at a ratio of 1:100 and loaded into a configured die and pressed at 20 MPa. The film thickness that was obtained after pressing was 0.4 mm. FTIR spectroscopy (Bruker Tensor, Kaller, Germany) analysis was performed to determine the interaction between proteins found in cell-free extract and SeNPs over a spectral range of 1000–4000 cm^−1^ [41]. A qualitative analysis of the spectral lines obtained from the test was conducted. 

Moreover, excessive concentration of sodium selenite may also destroy the integrity of strain cells. So, 50 mM selenite was selected to determine whether the concentration would destroy the integrity of strain Y4 cells. Scanning electron microscopy (SEM) was used for observing the distribution positions of bacteria and SeNPs produced by *B. paralicheniformis* Y4 under growth conditions.

#### 4.6.3. Stability of SeNPs at Different pH

Zeta potential values other than −30 mV to +30 mV (depending on the charge) are generally considered to have sufficient repulsive force whereby the nanoparticles to remain in the colloidal system [41]. On the other hand, a small zeta potential value can result in particle aggregation and flocculation due to the van der Waals attractive forces acting upon them [73]. Zeta Potential of the SeNPs produced by strain Y4 was measured using Zetasizer Nano Series (Malvern). SeNPs at pH 6, 7, 8, 9, and 10 were dispersed in H_2_O and sonicated for 10 min. A total of 1.5 mL of the suspension was then transferred to the cuvette of the dip cell kit for particle size distribution for zeta potential measurements. 

Determination of sedimentation rate was also conducted through Ultraviolet-visible Spectrophotometer. SeNPs at pH 3, 5, 7, 9, and 11 were dispersed in H_2_O and sonicated for 5 min. The optical density of the bacterial suspension at 420 nm was used to determine the sedimentation efficiency of the SeNPs.

### 4.7. Assay for the Probiotic Activity of Strain Y4 

#### 4.7.1. Extraction of Ethyl Acetate Extract of Strain Y4

Strain Y4 was grown in a 1 L LB medium for 48 h, and the cells were removed from the medium by centrifugation at 8000 rpm for 10 min. After extraction with ethyl acetate 1/1 (*v*/*v*), the supernatant was dried and weighed.

#### 4.7.2. α-Glucosidase Inhibitory Assay

The following solutions were prepared: 0.1 mol/L PBS (pH 6.8), 0.5 mmol/L PNPG solution, 0.3 U/mL α-glucosidase solution, 0.1 mol/L Na_2_CO_3_ solution, SeNPs, and ethyl acetate extract with concentrations of 10, 5, 2.5, 1, and 0.1mg/mL.

α-glucosidase inhibitory activity was assessed in 96-well plates using 0.01 M PBS (pH 7.0) buffer solution [74,75]. The assay was conducted in the 200 μL reaction system, which contained 25 μL PNPG, 25 μL samples, and 100 μL Na_2_CO_3_ solution. PNPG was 1/1 (*v*/*v*) mixed with the samples, incubated at 37 °C for 10 min, and then α-glucosidase solution was added and incubated at 37 °C for 30 min. Na_2_CO_3_ solution was added to terminate the reaction and the OD_405nm_ was determined. Acarbose was used as the positive control, and all assays were performed in three replicates. The inhibition rate was calculated using the following equation:

Inhibition rate (%) = (1 − (A − B)/(C − D)) × 100(1)

where A is the absorbance of the sample, B is the absorbance of sample blank control (α-glucosidase solution (replaced by PBS), C is the negative control absorbance (the sample is replaced by PBS), and D is the absorbance of negative blank control (sample and α-glucosidase solution was replaced by PBS).

#### 4.7.3. Antioxidant Evaluation of Ethyl Acetate Extract

The ethyl acetate extract of strain D1 was dissolved in ethanol to prepare a 10 mg/mL sample of mother liquor. The sample mother liquor was gradually added to reaction solutions with different volumes of 1, 5, 10, 20, and 30 μL. The final concentration of the sample was converted to 16, 80, 160, 320, and 480 μg/mL. 

##### ABTS Scavenging Capacity

The free radical scavenging activity of ethyl acetate extract was determined by ABTS radical cation decolorization assay according to the modified method of Re et al. [76]. ABTS was oxidized by potassium persulfate (K_2_S_2_O_8_) to its radical cation. For the assay, K_2_S_2_O_8_ was mixed with 20 mL of 7.4 mol/L ABTS solution (dissolved in distilled water) for 12 h at room temperature in the dark to yield a dark blue-green solution. The solution was diluted with H_2_O (pH 7.4) to attain an absorbance value of 0.75 at 734 nm and used for the antioxidant assay within 4 h. Samples were dissolved in water, and 70 µL of each sample solution was mixed with 600 μL of the diluted ABTS solution at 37 °C for 30 min, followed by a light absorbance measurement at 734 nm. The ABTS scavenging ability was calculated using the following equation:

DC/% = (1 − (As − Ac)/Ab) × 100(2)

where DC is the ABTS radical scavenging rate, As is the absorbance value of the sample mixed with ABTS working solution, Ac is the absorbance value after mixing the sample with water, and Ab is the absorbance value of ABTS working solution mixed with water.

##### DPPH Scavenging Capacity 

The reducing ability of ethyl acetate extract toward DPPH was measured according to the modified method of Brand-Williams et al. [77]. A volume of 600 μL of DPPH solution in methanol (0.25 mmol/L DPPH) was mixed with 70 μL of sample and 1.8 mL of distilled water. The mixture was shaken vigorously and incubated at room temperature for 30 min in the dark, and the absorbance at 517 nm was then measured and normalized to a blank sample. The DPPH scavenging ability was calculated using the following equation:

DC/% = (1 − (As − Ac)/Ab) × 100(3)

where DC is the DPPH radical scavenging rate, As is the absorbance value of the sample mixed with DPPH working solution, Ac is the absorbance value after mixing the sample with water, and Ab is the absorbance value of DPPH working solution mixed with water.

##### Hydroxyl Scavenging Capacity 

The hydroxyl radical scavenging activity of ethyl acetate extract was assessed according to the modified method of Brands S et al. [78]. A total of 200 μL of the ethyl acetate extract was supplemented with 200 μL of 9 mmol/L FeSO_4_·7H_2_O, 200 μL of 9 mmol/L salicylic acids, and 200 μL of 6 mmol/L H_2_O_2_. The mixture was incubated at 37 °C for 30 min before being applied to a spectrophotometer to measure the absorbance at 510 nm. The Hydroxyl scavenging capability was calculated using the following equation:

DC/% = (1 − (As − Ac)/Ab) × 100(4)

where DC is the hydroxyl radical scavenging rate, As is the absorbance value of the sample mixed with hydroxyl, Ac is the absorbance value after mixing the sample with water, and Ab is the absorbance value of hydroxyl mixed with water.

### 4.8. Determination of Environmental Adaptability of Strain Y4

Since temperature had a great influence on the growth of the microorganisms, this could affect the reduction rate of sodium selenite at different temperatures [79,80]. Therefore, our study determined temperature as the crucial factor in the growth of strain Y4. NaCl concentration and pH have a certain impact on the growth of the strain, so we also choose those two factors.

#### 4.8.1. Effects of Different Temperatures on the Growth of Strain Y4

Strain Y4 inoculum was added with 1 mL (1% *v*/*v*) to each triplicate tube of LB medium at 27, 30, 33, 34, 37, and 40 °C, and at 180 rpm. After 12, 24, and 36 h of incubation, samples were collected and the absorbance at 600 nm wavelength was measured. 

#### 4.8.2. Effects of Different pH on the Growth of Strain Y4

Strain Y4 grown in LB (37 °C, 12 h) was adjusted to pH 5, 6, 7, 8, and 9, and 1 mL (1% *v*/*v*) was added to each triplicate tube LB medium at 37 °C and 180 rpm. After 12, 24, and 36 h of incubation, samples were collected and the absorbance at 600 nm wavelength was measured. 

#### 4.8.3. Effects of Different NaCl Concentrations on the Growth of Strain Y4

Strain Y4 grown in LB (37 °C, 12 h) was added into 1%, 3%, 5%, 7%, and 9% NaCl solution and 1 mL (1% *v*/*v*) was added to each of the triplicate tubes of LB medium at 37 °C and 180 rpm. After 12, 24, and 36 h of incubation, samples were collected and the absorbance at 600 nm wavelength was measured.

### 4.9. CMCase Activity Assay Containing Selenite

Strain Y4 grown in LB without selenite and 5 mM selenite (37 °C, 12 h) was inoculated in LB liquid medium, and 1 mL (1% *v*/*v*) was added to the fermentation medium, incubated at 37 °C and 180 rpm for 48 h. The CMCase activity was measured by estimating the reducing sugars liberated from CMC. The enzyme assay was carried out by incubating the enzyme with CMC at 37 °C for 15 min. The reaction mixture (100 μL) contained 50 μL of enzyme and 1% (*w*/*v*) final concentration of CMC in 50 mM phosphate buffer (pH 7.0). The reducing sugar was estimated by the method of Singh S and Balasubramanian N with minor modifications [81,82]. The absorbance was measured at 540 nm using a UV-visible spectrophotometer (Perkin Elmer, Waltham, MA, USA, Model lambda-45) against a blank with D-glucose as standard. One unit (U) of cellulase activity is defined as the amount of enzyme that liberates 1 µmol of reducing sugar (glucose) in 1 min at 37 °C and pH 7.0. 

### 4.10. Statistical Analysis

All data from the present experiments were expressed as means ± standard deviation (SD) of three biological replications. SPSS Statistics software was used for statistical analysis. For studying the significant difference between the average, the different alphabetic superscripts in the same column are significantly different (*p* < 0.05) based on Tukey’s multiple comparison test.

## 5. Conclusions

At present, the protease-producing activity, genome analysis, depolymerization of the ramie fiber, and biological control of *Bacillus paralicheniformis* are mainly studied. However, the carboxymethyl cellulase activity of selenium-enriched *Bacillus* has not been observed. The analyses indicated that *B. paralicheniformis* Y4 can reduce selenite to SeNPs and generate high-yield CMCase activity in the presence of 5 mM selenite, which has not been previously reported. The maximum tolerance of *B. paralicheniformis* Y4 to selenite is 400 mM, which laid a foundation for the biotransformation of the SeNPs. The prepared SeNPs have an average particle size of 80 nm and zeta potential of-35.8 mV at a pH of 7.0, which has strong stability and is conducive to the industrial application of SeNPs as a feed additive.

Furthermore, the SeNPs and ethyl acetate extract have a potential inhibitory effect on α-glucosidase. The ethyl acetate extract of *B. paralicheniformis* Y4 has a certain DPPH and hydroxyl free radical scavenging ability and shows high free radical scavenging activities in ABTS. The CMCase activity was the highest at 8.96 U/mL after 72 h. *B. paralicheniformis* Y4 showed a high tolerance to selenite and environmental adaptability. The SeNPs have great stability and the addition of selenite could significantly improve the CMCase activity. As a potential candidate microorganism for the production and preparation of SeNPs as a feed additive, *B. paralicheniformis* Y4 has a practical use for industrial purposes. This can significantly improve the added value of agricultural products and achieve a win–win situation of economic development and environmental protection. However, further studies are required to formulate appropriate process parameters to transform the microbial strain for industrial production.

## Figures and Tables

**Table 1:** The selenium tolerance and the ratio of hydrolysis circle diameter of the 16 strains.

Stain	Selenium Tolerance (mM)	The Ratio of Hydrolysis Circle Diameter (d/D)
A1	200	2.75
B17	250	2.01
C8	250	2.64
Y4	400	3.23
SMJ-A8	200	3.10
SMJ-B9	300	2.93
SMJ-D11	250	1.97
SMJ-E13	300	2.31
SMJ-F20	200	2.84
XYT-A09	350	3.12
XYT-B15	200	3.02
XYT-B17	250	3.19
XYT-C03	300	2.93
XYT-D19	350	2.84
XYT-E12	250	2.71
XYT-G05	200	2.56

**Table 2:** Differential characteristics of strain Y4 and *Bacillus licheniformis*.

Characteristic	Strain	
	Strain Y4	*Bacillus licheniformis*
Voges-Proskauer test	+	+
L-arabinose	−	−
D-mannose	+	+
Sucrose	−	−
α-D-glucose	+	+
Lactose	−	−

+ Positive; − Negative.

**Table 3:** Free radical scavenging rate of ethyl acetate extract of strain Y4.

Item	The Concentration of Ethyl Acetate Extract (μg/mL)	The Free Radical Scavenging Rate (%)
ABTS	16	97.8 ± 0.87
DPPH	480	30.3 ± 2.8
Hydroxyl	320	78.5 ± 2.2

## Data Availability

The data is available on request.

## Sample Availability

The authors confirm that the data supporting the findings of this study are available within the article.
